# Exploring an optimal wavelet-based filter for cryo-ET imaging

**DOI:** 10.1038/s41598-018-20945-6

**Published:** 2018-02-07

**Authors:** Xinrui Huang, Sha Li, Song Gao

**Affiliations:** 1 School of basic medical sciences, Peking university, Department of Biophysics, Beijing, 100191 China; 2 School of foundational education, Peking university, Department of Medical Physics, Beijing, 100191 China

## Abstract

Cryo-electron tomography (cryo-ET) is one of the most advanced technologies for the *in situ* visualization of molecular machines by producing three-dimensional (3D) biological structures. However, cryo-ET imaging has two serious disadvantages—low dose and low image contrast—which result in high-resolution information being obscured by noise and image quality being degraded, and this causes errors in biological interpretation. The purpose of this research is to explore an optimal wavelet denoising technique to reduce noise in cryo-ET images. We perform tests using simulation data and design a filter using the optimum selected wavelet parameters (three-level decomposition, level-1 zeroed out, subband-dependent threshold, a soft-thresholding and spline-based discrete dyadic wavelet transform (DDWT)), which we call a modified wavelet shrinkage filter; this filter is suitable for noisy cryo-ET data. When testing using real cryo-ET experiment data, higher quality images and more accurate measures of a biological structure can be obtained with the modified wavelet shrinkage filter processing compared with conventional processing. Because the proposed method provides an inherent advantage when dealing with cryo-ET images, it can therefore extend the current state-of-the-art technology in assisting all aspects of cryo-ET studies: visualization, reconstruction, structural analysis, and interpretation.

## Introduction

Cryo-electron tomography (cryo-ET) is one of the most advanced technologies for the *in situ* visualization of molecular machines^1–3^. Cryo-ET produces three-dimensional (3D) views of single and unique biological objects, such as bacteria or cells, by imaging objects from a series of tilting angles and combining these images to produce a 3D reconstruction. Cryo-ET can provide valuable information regarding the structural basis of many cellular processes^4–6^. At present, the electron optical resolution in a cryo-image might be excellent; however, the resolution is limited as it depends on the sample thickness as well as the resistance of the sample to the applied beam^7^. A tolerable dose must be divided among the images taken at different angular orientations, and the dose per tilted view should be able to produce images with sufficient detail to enable their accurate alignment for back projection reconstruction^8^. Restrictions on the total dose and limitations in camera performance result in high noise levels that obscure the fine details in a cryo-tomogram^9,10^. Thus, the resolution of 3D density maps rarely exceeds 30 Å when using conventional cryo-ET methods. Therefore, developing a method to minimize noise and improve image resolution has become an important challenge in this field^10–15^.

The main purpose of this study is to explore an optimal wavelet transform (WT) method to reduce noise in a cryo-ET image. The WT has emerged as a powerful and efficient tool for data analysis, and it has shown potential in several applications such as noise filtering, image segmentation, and image compression^16–29^. Many parameters are used in WT to create different types of results that directly influence visual inspection and data analysis. Because there exist neither a systematic estimation method for evaluating image quality after wavelet denoising, nor empirical parameters for selection, we developed a quasi-systematic estimation method and determined a set of parameters for removing noise in cryo-ET images.

In this paper, we describe our proposed method for obtaining optimal wavelet parameters based on our four types of designed wavelet filters, and we present their tested performance capabilities based on simulation data. Then, we compare a wavelet filter designed using optimal parameters against undenoised results based on cryo-ET experiment data. Finally, we present the discussion and some concluding remarks.

## Results

### Results of testing with simulation data

In this section, we present visual and quantitative results filtered using our 2D or 3D wavelet filters when applying them to cryo-ET visualization and reconstruction.

### Visualization

The images processed according to different denoising strategies are shown in Supplementary Fig. S2. It is clearly visible that background noise is decreased as compared to the original noisy data in Supplementary Fig. S1(B).

For the direct soft-thresholding denoising strategy (“w1”), the result of the 2D wavelet filter does not seem to have any significant difference compared with that of the 3D wavelet filter. For the modified shrinkage denoising strategy (“w2”), when zeroed out, the decomposition levels were set to 1, (1, 2), or (1, 2, 3), and the background noise significantly decreased and a very good contrast was achieved. Moreover, when compared with zeroing out the decomposition levels of (1, 2) or (1, 2, 3), when decomposition level 1 was set to zero, the edge and detailed structure were better preserved, and the other two decomposition-level selection methods clearly produced excessive blurring. The results from similar decomposition-level selection methods showed a similar phenomenon for spatially adaptive (SA) thresholding (“w3”). For cross-scale regularization (CSR) (“w4”), when decomposition level (1, 2) was modified, better performance was achieved than when only modifying decomposition level 1, although the results were slightly worse than the best results shown in “w2” and “w3”. When the same decomposition level changed, 3D filters performed better than 2D filters. These phenomena were also seen in “w2” and “w3” when decomposition level 1 was selected for modification.

For a global analysis, Supplementary Fig. S3 shows the signal-to-noise ratio (SNR), mean-squared error (MSE), and cross-correlation coefficient (CCC) comparisons of the WT-processed images. The quantitative evaluation results from Supplementary Fig. S3 agree well with visual evaluation shown in Supplementary Fig. S2. These results also indicate that, when decomposition level 1 was changed for both the modified shrinkage and SA thresholding denoising strategies, the filters can preserve the details and texture, or achieve stronger noise smoothing and more pronounced contrast for the most prominent features, particularly for the 3D case. The results can be visually compared with the noise-free and noisy phantoms in Supplementary Fig. S1 and be quantitatively compared with SNR, MSE, and CCC of “noisy” in Supplementary Fig. S3. Compared with “w2_1” or “w3_1”, better visual results have higher SNR values, lower MSE values, and higher CCC values.

### Reconstruction

Supplementary Fig. S4 indicates that the use of Simultaneous Iterative Reconstruction Technique (SIRT) provides better results than using Weighted Back Projection (WBP). This is because the two main limitations of electron tomography are a missing wedge and the low SNR of the images (which is the case with cryo-ET). The robustness of the WBP and SIRT results achieved by our filters with these four denoising strategies was evaluated. A comparison of the 3D reconstructions computed using the filtered tilt series to the original noise-free phantom was conducted using the SNR, MSE, and CCC. Running TomoJ^30^ on the noisy projections of the phantom shown in Supplementary Fig. S1, we obtained the 3D reconstruction models for the WBP and SIRT methods (shown in “noisy”). Different filters may be preferred depending on which decomposition level is modified. Compared to the orthogonal visual results of the 3D reconstruction models, the modified shrinkage method for zeroed-out decomposition level 1 was found to achieve the best tradeoff between noise suppression and detail preservation visually, as shown in “w2”. In addition, under this situation, the WBP result was apparently improved owing to noise suppression. The SIRT result appeared to be mildly improved. The best result of SA thresholding, shown in “w3”, is very similar to that shown in “w2”, in terms of quality. Supplementary Fig. S5 shows the comparison of the SNR, MSE, and CCC between “w2_1” and “w3_1”, which indicates that the modified shrinkage filter provides a better result, and it clearly illustrates that noise reduction enhances the visual reconstruction of an image.

### Results of testing with experiment data

In this section, we present the visual results of filtering real cryo-ET experiment data using our 2D or 3D optimal wavelet filters, and we provide the comparison results against undenoised results.

### Visualization

**Figure 1:**
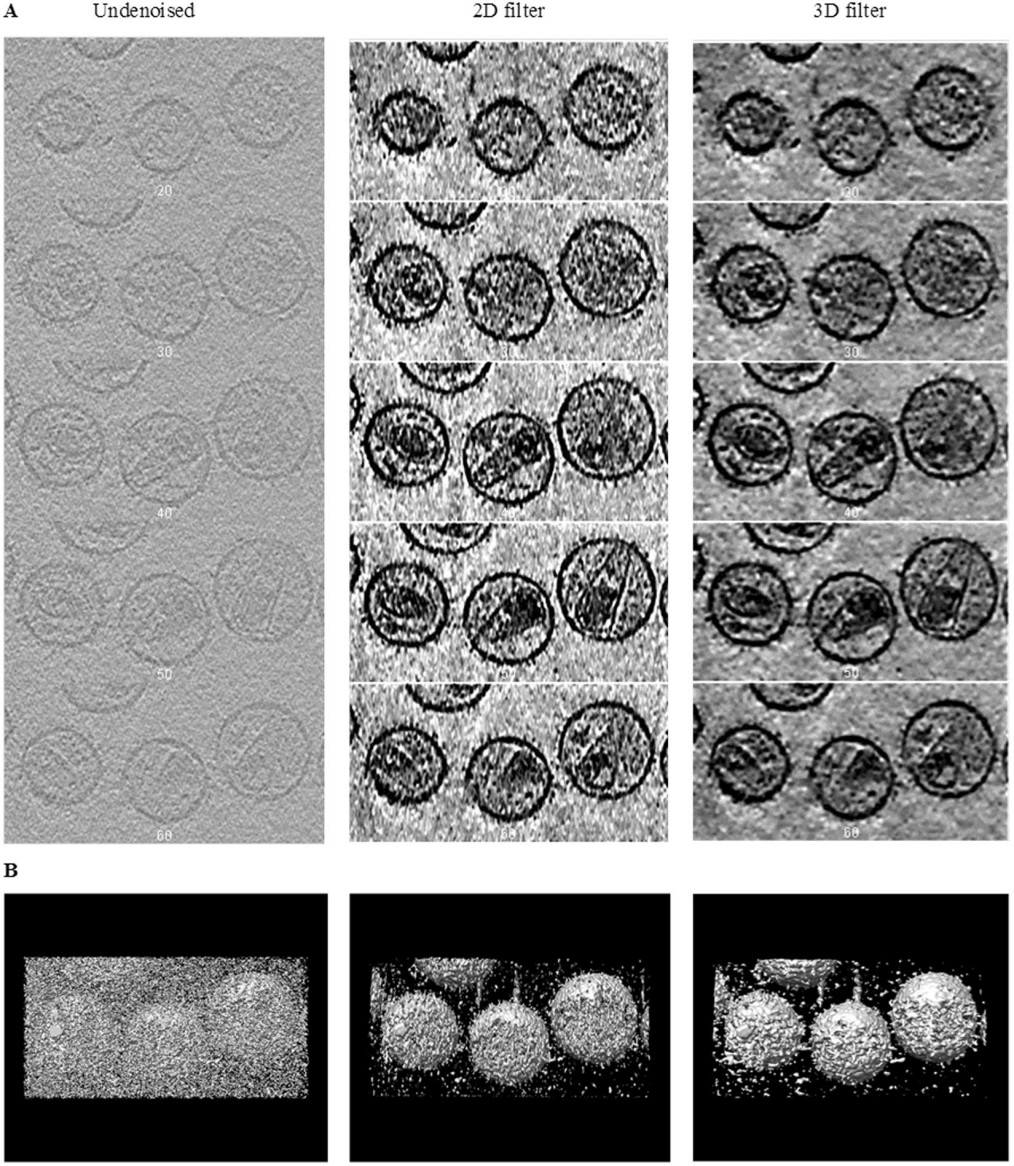
Comparison of denoising the cryo-tomogram using the optimal 2D/3D filter. (**A**) Z slices montage; (**B**) 3D view.

As shown in Fig. 1A, the selected Z-slices of the volume data processed using our optimal wavelet filter show significantly more contrast than the original undenoised cryo-tomogram, and significantly enhanced virion features; in addition, most of the noise is eliminated, and the contrast is enhanced to a level at which a meaningful surface representation can be rendered (Fig. 1B). The 3D wavelet filter performed better than the 2D wavelet filter as indicated by the test results with the simulation data.

### Reconstruction

**Figure 2:**
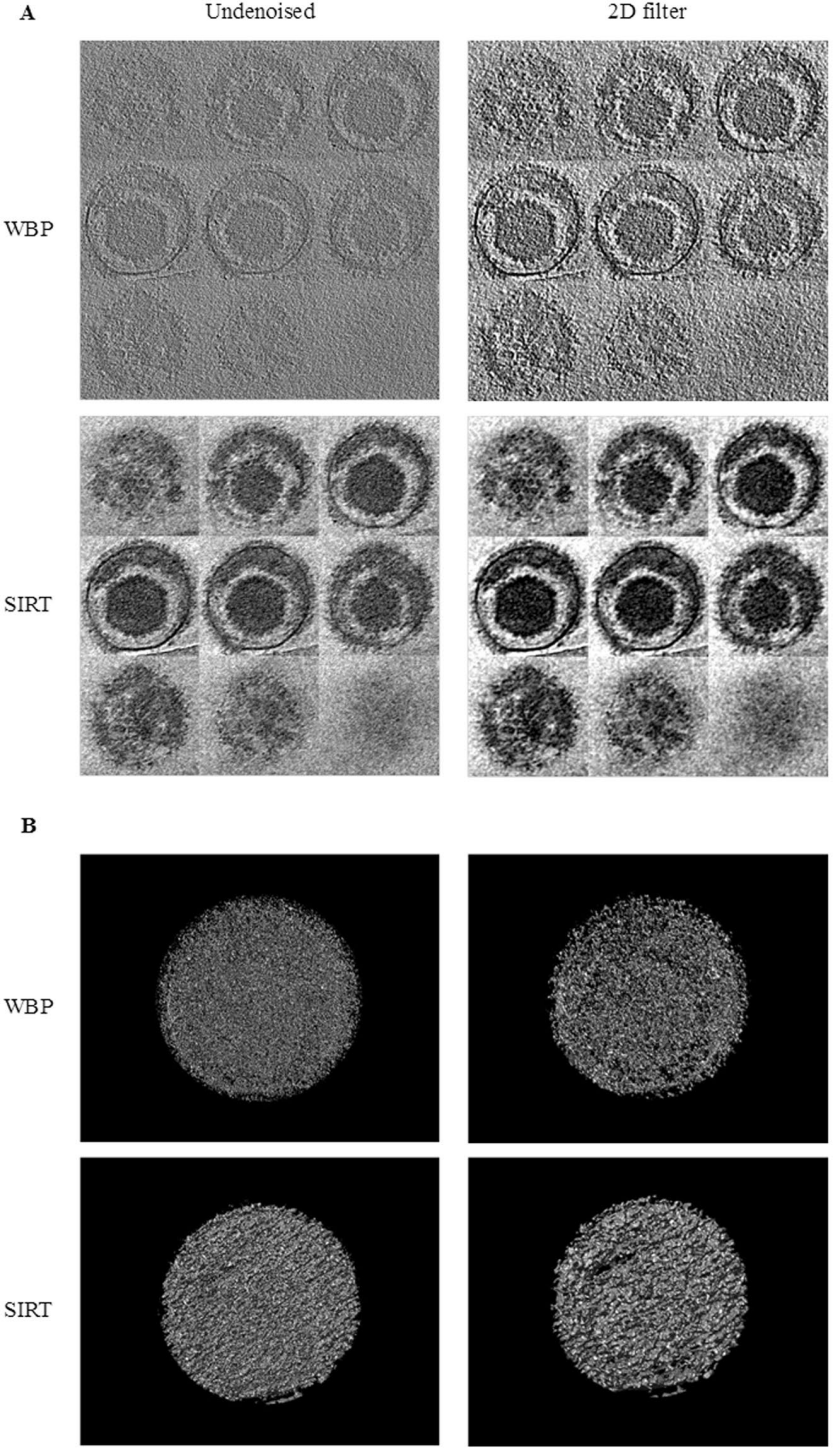
Comparison of WBP and SIRT reconstructed maps with cryo-ET tilt series undenoised and denoised by the optimal 2D wavelet filter. Different display styles are used to show the reconstructed maps: (**A**) Grouped Z-slices; (**B**) 3D View.

In Fig. 2A, a gallery of parallel slices along the Z-axis is shown. Each slice represents the average over the grouping of seven Z planes, called “Grouped Z-slices”. Fig. 2B shows the virion framed in a “3D View” with the UCSF Chimera.

As shown in Fig. 2A with “Grouped Z-slices”, the optimal wavelet filter performs better in terms of image contrast, as well as texture preservation and edge preservation. Three distinctive spike morphologies, i.e., bifurcated spikes, spikes with an emergence angle, and curved spikes, are clearly shown in the denoised tomogram when applying optimal wavelet filter preprocessing. In addition, some spikes with tegument densities inside the envelope are in close contact. The transmembrane contact between a glycoprotein and tegument can also be seen. An intraviral nucleocapsid structure with icosahedral symmetry appears clearly. Some pentons are marginally visible. As shown in the “3D View” for a single virion denoised tomogram, the outer surface shows a distribution of glycoprotein spikes protruding from the membrane. For all results described above using the optimal wavelet filter, the SIRT results are better than the WBP results, which are similar to the simulation data.

## Discussion

The results show that wavelet filters have the potential to improve both the high-resolution information and contrast of cryo-tomograms. We provided a review of wavelet-based denoising methods for cryo-ET together with a performance evaluation. Different objective performance measures were assessed. The results of the presented objective performance analysis agreed well with the visual assessment. The wavelet filter with optimum parameters (three-level decomposition, level-1 zeroed out, subband-dependent threshold, a soft thresholding, and spline-based discrete dyadic wavelet transform (DDWT)) was then selected, and we called it the modified wavelet shrinkage filter. Many parameters were selected for wavelet denoising; in our experiments, we used the DDWT, which is more effective in terms of noise reduction and maintains relatively complete data after image decomposition. In addition, we used three levels for the decomposition because processing three levels obtains a better noise removal effect when compared to one or two levels. Soft thresholding was selected to reduce the variance (noise) in the images because hard thresholding is discontinuous and yields abrupt artifacts in the denoised images, particularly when the noise energy is significant^31^. The selection of a thresholding method directly influences how much noise is to be removed at each level. In our study, we evaluated the outputs of different threshold selection methods, i.e., simple constant threshold application, universal threshold and subband-dependent threshold estimation, and SA thresholding. Our experiments show that SA estimation generates relatively the same results as subband-dependent threshold estimation visually; however, subband-dependent thresholding is slightly better. Subband-dependent thresholding prevails in terms of its computational simplicity.

As we know, the main procedure for processing a cryo-ET tilt series includes alignment, reconstruction, and visualization. Using this 2D optimal wavelet filter to preprocess different tilt angle 2D images in a real tilt series of cryo-ET, the displacement (translational errors) and measuring tilt errors (including tilt-axis and tilt-angle errors) can be reduced during the alignment step. Using the same tomography reconstruction methods, WBP and SIRT, a filtered tilt series provides a better resolution, especially for the WBP method. This is because this new strategy largely avoids noise to disturb computation and can precisely determine/correct such tilt-related errors and translational errors. For the 3D reconstructed maps, after applying the 3D optimal wavelet filter to postprocess the entire volume, the filtered volume provides better visualization. Therefore, combining 2D and 3D wavelet filters to process cryo-ET data can contribute to high-resolution cryo-ET reconstruction maps.

In general, spatial filtering methods have the disadvantage of losing image resolution when reducing noise. From the above performance of the modified wavelet shrinkage filter, we observe that wavelets can allow for joint resolution in both the spatial and frequency domains. The wavelets’ basis functions are of finite duration and varying scale, making it possible to examine signals at differing resolutions. Thus, the modified wavelet shrinkage filter can offer a remarkable improvement in image quality with a good compromise between detail preservation and noise smoothing. We expect that our study can provide benefits to cryo-ET applications and the interpretation of biological structures.

## Methods

### Wavelet-based filters

Wavelet decomposition of a signal is based on a collection of compactly supported oscillatory basis functions that are related to each other through scaling and translations. The inclusion of localized fine-scale functions in a basis allows significant details to be better obtained at multiple scales. Based on the multiresolution analysis (MRA) and the discrete wavelet transform (DWT) theory^32,33^, our noisy cryo-ET image *f*(*n*), (where *n*∈{1, 2, … *N*} is a single lexigraphic index that varies over all *N* pixels in the image), can be represented as

1 $$f(n)=\frac{1}{\sqrt{N}}\sum _{m}S[J,m]{\phi }_{J,m}(n)+\frac{1}{\sqrt{N}}\sum _{j=1}^{J}\sum _{m}W[j,m]{\psi }_{j,m}(n).$$

The first sum provides a lower resolution approximation of *f*(*n*), and the second sum represents a set of increasingly fine details that are added to the low-resolution approximation.

The DWT at the scale *J* consists of sets of approximation coefficients {*S*[*J*, *m*]}_*m*∈*Z*_ and detail coefficients {*W*[*j*, *m*]}_*m*∈*Z*,*j*∈[1,*J*]_.

2 $$S[J,m]=\frac{1}{\sqrt{N}}\sum _{n}\,f(n){\phi }_{J,m}(n),$$

3 $$W[j,m]=\frac{1}{\sqrt{N}}\sum _{n}\,f(n){\psi }_{j,m}(n),$$

where *φ*_*J,m*_(*n*) and *ψ*_*J,m*_(*n*) are discretized versions of the scaling and wavelet functions.

**Figure 3:**
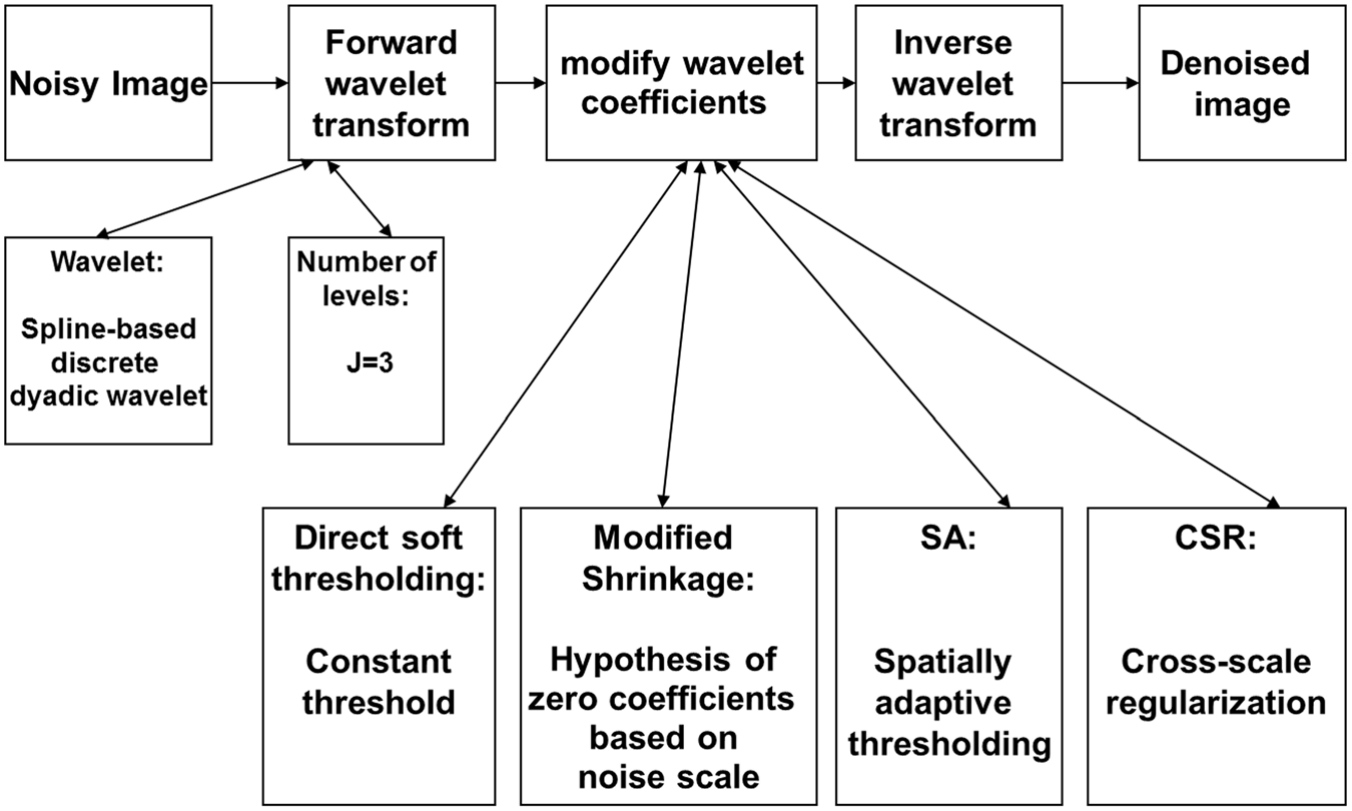
Flowchart of exploring an optimum wavelet filter.

Assuming noisy cryo-ET image *f*(*n*) with white Gaussian noise,

4 $$f(n)=I(n)+\varepsilon (n)$$

where *I*(*n*) is a “true” underlying image that we are trying to recover; *ε(n)* is a noise contamination term that we are trying to remove; and the goal of image denoising is to obtain *f*_*denoised*_, which is an estimate of *I* based on noisy observation *f*, and any known properties of *ε* (ε ~ *Norm*(0, *σ*^2^), where *σ*^2^ is the noise variance in noisy image *f* ). In this work, the denoising procedure applied as given in the flowchart in Fig. 3.

Step 1: Apply a D-dimensional, three-level, spline-based DDWT to noisy image *f* (where D = 2 is a 2D wavelet filter, and D = 3 is a 3D wavelet filter).

A DDWT is a powerful tool in signal analysis and processing, and it belongs to the class of redundant frame expansion of signals, exhibiting translation invariance (overcoming orthogonal wavelet transforms), and very good multiresolution analysis capabilities at the expense of undecimation^34^. Koren and Laine systematically extended the spline-based DDWT theory (and its filter bank implementation) to more than two dimensions, realizing that such a representation can be advantageous in medical imaging applications^35^. Because biomedical signals are commonly characterized as having smooth transitions as opposed to harsh jump discontinuities, we use a spline-based DDWT in our study. The D-dimensional DDWT implementation uses a filter bank structure with a three-level decomposition, i.e., *J* = 3. After implementing this step, we can obtain wavelet detail coefficients {*W*[*j, m*]}_*m*∈*Z,j*∈[1,*J*]_, where *m* is an index over all detail coefficients at all decomposition levels. Considering the D-dimensional style, the wavelet detail coefficients here are described as ${}^{d}W_{j}[{m}_{1},{m}_{2},\ldots ,{m}_{D}]$, where ^*d*^*W*_*j*_ indicates the detail coefficients in the subband being processed at the decomposition level *j*, dimension *d*, where *j* = 1, 2, 3; *d* = 1, 2, …, *D*. Here, *m*_*d*_ is an index over all detail coefficients in dimension *d*.

Step 2: Apply four types of denoising strategies to modify wavelet detail coefficients ^*d*^*W*_*j*_.

I. Direct soft thresholding
  A number of wavelet-based denoising algorithms are based on *wavelet thresholding*, a simple and effective technique introduced by Donoho and Johnstone^36^. In general, two types of thresholding methods are used: soft thresholding and hard thresholding^31^. We chose soft thresholding (described later) owing to the latter’s jump discontinuity at the threshold value.
  5 $${}^{d}\tilde{W}_{j}=\{\begin{array}{ccc}0 & if & |{}^{d}W_{j}| < \lambda \\ {}^{d}W_{j}-\lambda  & if & {}^{d}W_{j}\ge \lambda \\ {}^{d}W_{j}+\lambda  & if & {}^{d}W_{j}\le -\lambda \end{array}$$
  Here, *λ* is a global constant or a universal threshold, i.e., $\lambda =\sigma \sqrt{2\,\mathrm{ln}\,N}$, where *σ* is the standard deviation (SD) of the measured data *f*.
II. Modified shrinkage
  First, the decomposition level(s) to be omitted, if any, are selected. We investigated different ways to modify the detail coefficients ${}^{d}W_{j}[{m}_{1},{m}_{2},\ldots ,{m}_{D}]$by selectively setting them to zeros to suppress the noise contribution in the wavelet domain at various decomposition levels, and assessed the effects that such methods have on the denoised output.
  Second, we used a subband-dependent threshold, i.e.,
  6 $${}^{d}\lambda _{j}[{m}_{1},{m}_{2},\ldots ,{m}_{D}]=\frac{{\sigma }_{j}^{2}}{{}^{d}\sigma _{j}[{m}_{1},{m}_{2},\ldots ,{m}_{D}]},$$
  where ${}^{d}\sigma _{j}[{m}_{1},{m}_{2},\ldots ,{m}_{D}]$ is the SD of the detail coefficients in a given subband. When *σ*_*j*_ is not explicitly available, it is usually unknown *a priori* and needs to be estimated from the detail coefficients *W*_*j*_ at the decomposition level *j*. A robust median estimator developed by Donoho and Johnstone can be used, i.e.,
  7 $${\hat{\sigma }}_{j}={\rm{Median}}(abs({W}_{j}[i]))/0.6745,$$
  where *i* is an index over all the detail coefficients *W*_*j*_.
  Finally, apply soft thresholding for each ^*d*^*W*_*j*_ with each ^*d*^*λ*_*j*_ using equation (5).
III. Spatially adaptive (SA) thresholding
  First, select the decomposition level(s), if any, to be modified using SA thresholding.
  Second, the thresholding adaptively varies within a subband of detail coefficients:
  8 $${}^{d}\tilde{W}_{j}[{m}_{1},{m}_{2},\ldots ,{m}_{D}]=\frac{1}{M}(\sum _{[{m}_{1},{m}_{2},\mathrm{...},{m}_{D}]\in M}{}^{d}W_{j}[{m}_{1},{m}_{2},\ldots ,{m}_{D}]),$$
  where *M* is a neighborhood of *M* pixels around the currently processed coefficient. For this study, *M* was selected as a 5 × 5 (for 2D) or 5 × 5 × 5 (for 3D) neighborhood, which was determined empirically.
IV. Cross-scale regularization (CSR)

First, select the decomposition level(s), if any, to be modified using CSR.

Second, modify the detail coefficients ^*d*^*W*_*j*_ using CSR. Using this algorithm, a coefficient modification is applied using the wavelet modulus instead of separately on the individual wavelet vector components. This is conducted to preserve the direction of the wavelet vector *w* (the gradient direction), where the signal changes most rapidly.

- At each coefficient position, compute the wavelet modulus of level *j* as
  9 $${M}_{j}[{m}_{1},{m}_{2},\ldots ,{m}_{D}]=\sqrt{\begin{array}{c}{({}^{1}W_{j}[{m}_{1},{m}_{2},\ldots ,{m}_{D}])}^{2}\\ +{({}^{2}W_{j}[{m}_{1},{m}_{2},\ldots ,{m}_{D}])}^{2}\\ +\ldots +{({}^{D}W_{j}[{m}_{1},{m}_{2},\ldots ,{m}_{D}])}^{2}\end{array}},$$
  and the unit vector defining its direction as
  10 $$\begin{array}{lll}{w}_{j}[{m}_{1},{m}_{2},\ldots ,{m}_{D}] & \mathop{=}\limits^{{\rm{\Delta }}} & \frac{1}{{M}_{j}[{m}_{1},{m}_{2},\ldots ,{m}_{D}]}[{}^{1}W_{j}[{m}_{1},{m}_{2},\ldots ,{m}_{D}]\\  &  & \times {}^{2}W_{j}[{m}_{1},{m}_{2},\ldots ,{m}_{D}]\ldots {}^{D}W_{j}[{m}_{1},{m}_{2},\ldots ,{m}_{D}],\end{array}$$
  b. Calculate the wavelet modulus at the next coarsest level (*j* + 1) for each coefficient position and normalize it to within the range [0, 1] by dividing the maximum value of the wavelet modulus at level (*j* + 1):
  11 $${M}_{j+1}^{norm}[{m}_{1},{m}_{2},\ldots ,{m}_{D}]=\frac{{M}_{j+1}[{m}_{1},{m}_{2},\ldots ,{m}_{D}]}{\max \{{M}_{j+1}\}}.$$
  Note that if the coefficients at level (*j* + 1) are being modified as part of the denoising protocol, the modification should be completed before applying this step.
- Find the modified wavelet modulus of the level *j* coefficients, ${\tilde{M}}_{j}[{m}_{1},{m}_{2},\ldots ,{m}_{D}]$, through the following pointwise multiplication:

12 $${\tilde{M}}_{j}[{m}_{1},{m}_{2},\ldots ,{m}_{D}]={M}_{j+1}^{norm}[{m}_{1},{m}_{2},\ldots ,{m}_{D}]\cdot {M}_{j}[{m}_{1},{m}_{2},\ldots ,{m}_{D}].$$

d. Generate the modified detail coefficients at each position by projecting the vector defined by ${\tilde{M}}_{j}$ and *w*_*j*_ onto dimension *d* as

13 $${}^{d}\tilde{W}_{j}[{m}_{1},{m}_{2},\ldots ,{m}_{D}]={\tilde{M}}_{j}[{m}_{1},{m}_{2},\ldots ,{m}_{D}]\cdot {w}_{j}[{m}_{1},{m}_{2},\ldots ,{m}_{D}].$$

Step 3: Apply the inverse DDWT based on modified wavelet coefficients ${}^{d}\tilde{W}_{j}$ to obtain the denoised image *f*_*denoised*_.

### Testing using simulation data

The simulation data was generated from the TomoJ phantom^30^, and a follow up analysis is provided. To compare the performance of the denoising methods, we visually inspected the filtered images and some gray-level profiles. For a global analysis, we also used a quantitative approach based on several figures of merit; for example, to evaluate the degree of noise reduction, the SNR defined using equation (14), the MSE defined using equation (15), and the CCC defined through equation (16), were calculated.

14 $$SNR=\frac{\sum _{n=1}^{N}{(I(n)-\bar{I}(n))}^{2}}{\sum _{n=1}^{N}{({f}_{denoised}(n)-I(n))}^{2}},$$

15 $$MSE=\frac{1}{N}\sum _{n=1}^{N}{({f}_{denoised}(n)-I(n))}^{2},$$

16 $$CCC=\frac{\sum _{n=1}^{N}({f}_{denoised}(n)-{\overline{f}}_{denoised}(n))(I(n)-\bar{I}(n))}{\sqrt{(\sum _{n=1}^{N}{({f}_{denoised}(n)-{\overline{f}}_{denoised}(n))}^{2})(\sum _{n=1}^{N}{(I(n)-\bar{I}(n))}^{2})}},$$

where *N* is the total number of values in the denoised data *f*_*denoised*_, and *I* is the noise-free data. ${\overline{f}}_{denoised}$ indicates the mean of the values in *f*_*denoised*_, and $\bar{I}$ indicates the mean of the values in *I*. Indeed, better denoised data will tend to give a higher SNR value and a lower MSE value. The CCC is nearer to 1 when the objects are more similar, and nearer to zero when they are more different.

### Visualization

We added Gaussian noise (SNR = 0.1, which is almost consistent with the noise level in a real cryo-tomogram) to the noise-free 3D phantom data and then filtered the noisy volume data by changing the processing parameters (Supplementary Fig. S1(A,B)). The 3D noisy phantom data were denoised using 2D and 3D wavelet filters separately. In the 3D case, the entire volume of data underwent 3D denoising concurrently. In the 2D case, each y-axis-oriented slice underwent 2D denoising, and this process was repeated independently for all the slices in the volume. The results were visualized using Chimera from UCSF^37^. The SNR, MSE, and CCC between the denoised and noise-free data were calculated using a suitable mask.

### Reconstruction

According to the data collection strategy of real cryo-ET imaging, the projection images of the 3D noise-free phantom data were obtained with a tilt angle range of [−70°, 70°], with 1° angle increments and vertical tilt axis (Supplementary Fig. S1(A,C)). Then, Gaussian noise (SNR = 0.1, which is almost consistent with the noise level in a real cryo-ET tilt series) was added to the noise-free tilt series to generate a noisy tilt series (Supplementary Fig. S1(D)).

Each angle-oriented projection image underwent 2D wavelet denoising. This process was repeated independently for all projection images within the tilt series. The undenoised and denoised tilt series were reconstructed using the WBP and SIRT algorithms, respectively. TomoJ was used as the reconstruction software. The SNR, MSE, and CCC between the reconstructed phantom and the noise-free phantom were calculated using a suitable mask.

### Testing using experiment data

We then used the above selected optimal wavelet parameters to process the real cryo-ET experiment data and compared the effectiveness with the undenoised results. Denoised images were visually inspected to assess whether the method significantly changed some of the global properties, such as the shape, size, and position of the relevant structures in the images.

### Visualization

For denoising a cryo-tomogram, we used a tomogram containing three authentic, unstained, ice-embedded HIV-1 virions. The data was obtained from the EM data bank (EMDB ID: EMD-1155). The HIV-1 cryo-tomogram was denoised using 2D and 3D filters separately. The results were compared and assessed through visualization.

### Reconstruction

A set of HSV-1 cryo-ET tilt series was used for the test, and was collected automatically at 1.5° increments over an angular range of [−63°, 60°] on FEI CM120 microscopes. The accelerating voltage was 120 kV, and the total electron doses were approximately 35 electrons/Å^2^. The undenoised and denoised tilt series were reconstructed using Inspect3D software from FEI, Inc. The results were compared and assessed through visualization.

### Software and data availability

All the data mentioned above and all the results are available. All the programs are coded in MATLAB software based on some researcher’s pioneer work. Thus, all our programs are open source and coded in MATLAB R2014a (http://www.mathworks.com/products/matlab/). Please freely download from our webpage (http://mpe.bjmu.edu.cn/CryoET/).

## Electronic supplementary material

Supplementary information
