# Supplementary material for: Exploring an optimal wavelet-based filter for cryo-ET imaging
Source: Sci Rep. 2018 Feb 7;8:2582. doi: 10.1038/s41598-018-20945-6 (PMC5803242; doi:10.1038/s41598-018-20945-6)
Supplement: Supplementary file 1 — Supplementary information [file 41598_2018_20945_MOESM1_ESM.pdf]

Supplementary Data

Exploring an optimal wavelet-based filter for cryo-ET imaging

Xinrui Huang[1], Sha Li[2], Song Gao[2,*]

[1] School of basic medical sciences, Peking university, Department of Biophysics, Beijing, 100191, China

[2] School of foundational education, Peking university, Department of Medical Physics, Beijing, 100191, China

[*]gaoss@hsc.pku.edu.cn

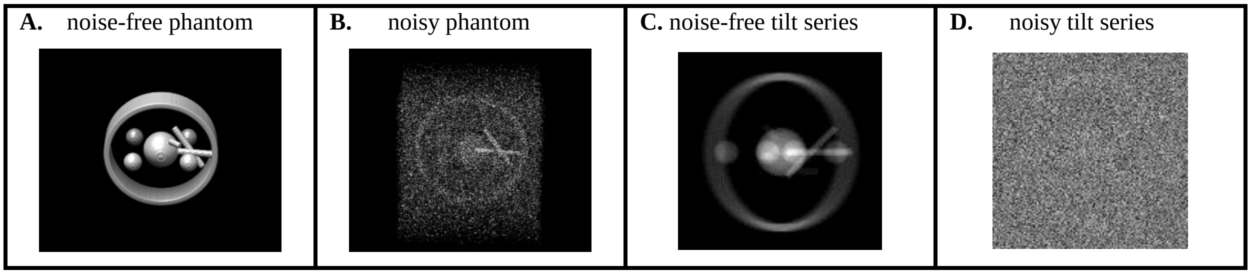

Figure S1. Simulation data used by exploring optimum wavelet parameters for cryo-ET visualization and reconstruction: (A) 3D noise free phantom; (B) 3D noisy phantom with noise(SNR=0.1) added to phantom in A; (C) noise-free tilt series generated with phantom showed in A; (D) noisy tilt series with noise(SNR=0.1) added to tilt series in C.

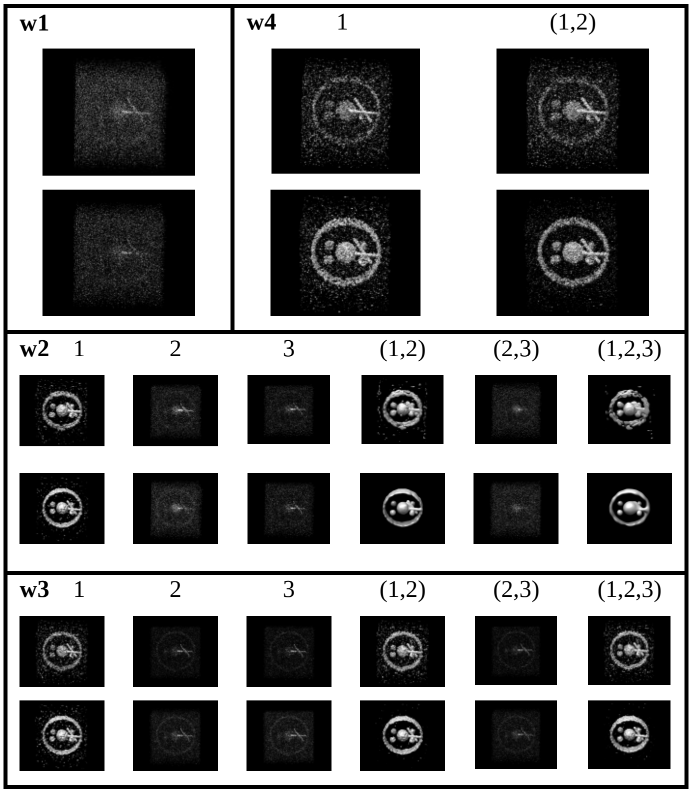

Figure S2. 3D view visual comparison of different denoising schemes for noisy phantom data with SNR = 0.1(showed in Figure S1(B)). Four types of denoising strategies applied for filtering noisy phantom in 2D (results showed in the first row on each figure) and 3D (results showed in the second row on each figure) style separately: "**w1**" represents direct soft thresholding with constant threshold 0.1;

“**w2**” modified shrinkage with different decomposition levels selected; “**w3**” SA thresholding with different decomposition levels selected; and “**w4**” CSR with different decomposition levels selected.

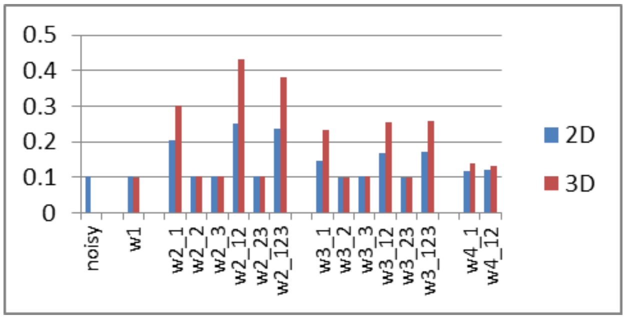

SNR

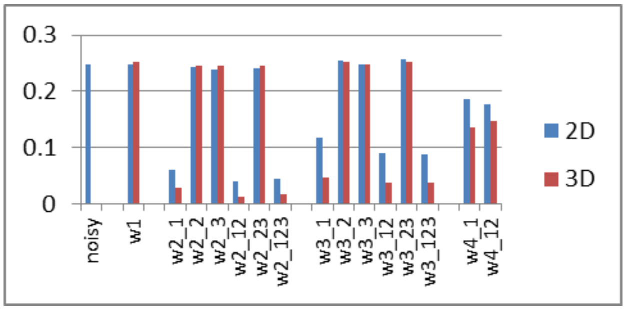

MSE

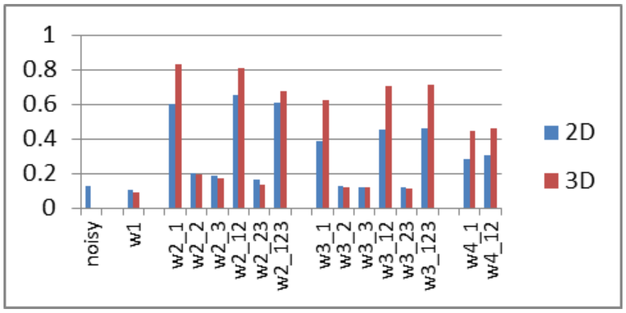

CCC

Figure S3. SNR, MSE and CCC comparison of different denoising schemes for noisy phantom data with SNR=0.1, corresponding to results showed in Figure S2.

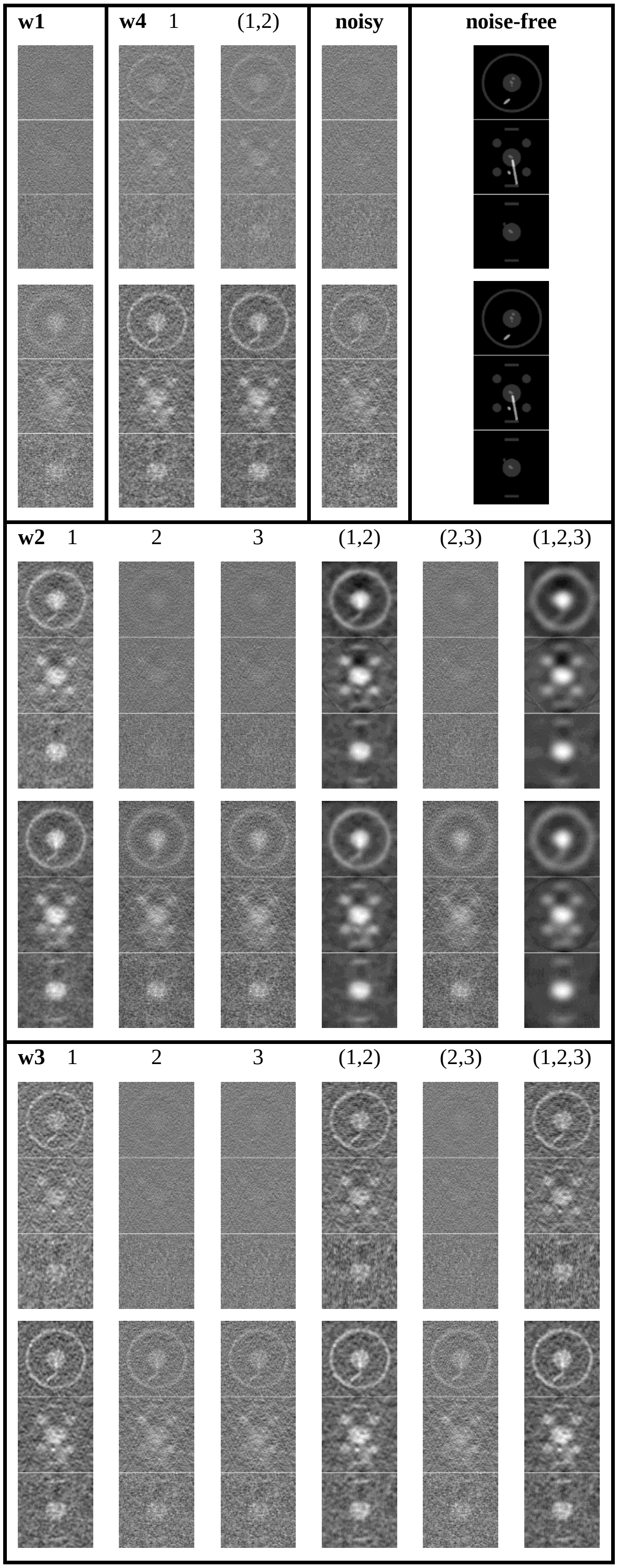
w1
w4
1
(1,2)
noisy
noise-free
w2
1
2
3
(1,2)
(2,3)
(1,2,3)
w3
1
2
3
(1,2)
(2,3)
(1,2,3)

Figure S4. Orthogonal views visual comparison of results reconstructed by preprocessing noisy tilt series of SNR = 0.1(showed in Figure S1(D) with different denoising schemes. Four types of denoising strategies were applied separately to provide the denoised tilt series used in WBP (results showed in the first row on each figure) and SIRT (results showed in the second row on each figure) reconstruction: "**w1**" represents direct soft thresholding with constant threshold 0.1; "**w2**" represents modified shrinkage with different decomposition levels selected; "**w3**" represents SA thresholding with different decomposition levels selected; "**w4**" represents CSR with different decomposition levels selected ; "**noisy**" represents noisy tilt series directly used in reconstruction, and "**noise-free**" represents noise free phantom results.

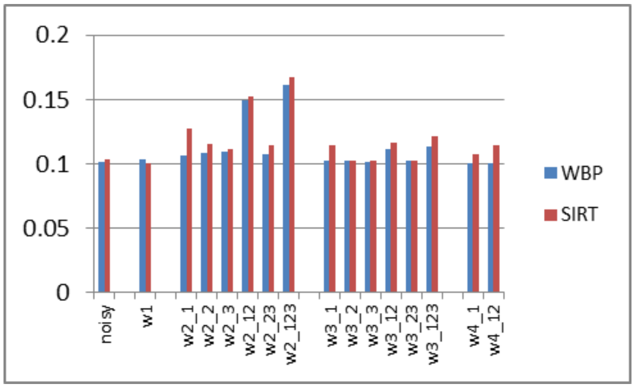

SNR

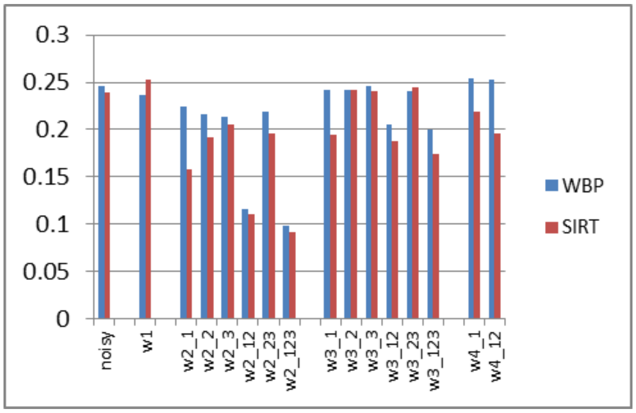

MSE

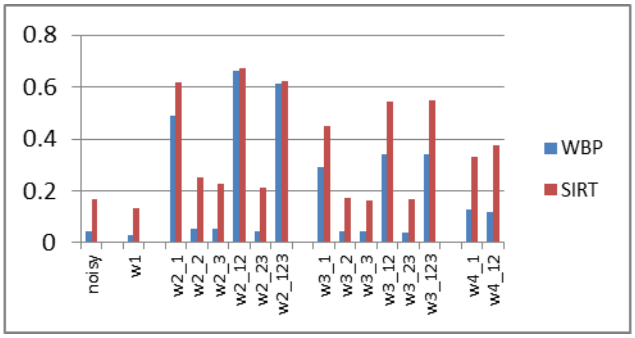

CCC

Figure S5. SNR, MSE and CCC comparison of results reconstructed by preprocessing noisy tilt series of SNR = 0.1 with different denoising schemes, corresponding to results showed in Figure S4.
